# Supplementary material for: The Genetic Architecture of Post-Zygotic Reproductive Isolation Between Anopheles coluzzii and An. quadriannulatus
Source: Front Genet. 2020 Aug 14;11:925. doi: 10.3389/fgene.2020.00925 (PMC7480394; doi:10.3389/fgene.2020.00925)
Supplement: Supplementary file 2 [file Table_1.docx]

**Table S1**. The complete list of annotated genes within autosomal QTL and their PANTHER gene ontology families / sub-families (if available).

| **QTL** | **Gene Name** | **Panther Gene Ontology Family / Subfamily** |
| --- | --- | --- |
| 2R A | AGAP001195 | NUCLEAR CAP-BINDING PROTEIN SUBUNIT 1 (PTHR12412:SF2) |
| 2R A | AGAP001226 |  |
| 2R A | AGAP001189 | GEO07581P1-RELATED (PTHR11857:SF43) |
| 2R A | AGAP001207 | COMM DOMAIN-CONTAINING PROTEIN 4 (PTHR16231:SF4) |
| 2R A | AGAP001202 | FI24011P1-RELATED (PTHR24064:SF335) |
| 2R A | AGAP001206 |  |
| 2R A | AGAP013230 |  |
| 2R A | AGAP013386 | DNA POLYMERASE ZETA CATALYTIC SUBUNIT (PTHR45812:SF1) |
| 2R A | AGAP001215 | TETRATRICOPEPTIDE REPEAT PROTEIN 39C (PTHR31859:SF1) |
| 2R A | AGAP001199 | AT20289P-RELATED (PTHR24276:SF78) |
| 2R A | AGAP001196 | G KINASE-ANCHORING PROTEIN 1 (PTHR14899:SF0) |
| 2R A | AGAP001200 | GLYCOGEN DEBRANCHING ENZYME (PTHR10569:SF2) |
| 2R A | AGAP013011 | TRICHOPLEIN KERATIN FILAMENT-BINDING PROTEIN (PTHR31183:SF2) |
| 2R A | AGAP013543 |  |
| 2R A | AGAP001217 | SOLUBLE CALCIUM-ACTIVATED NUCLEOTIDASE 1 (PTHR13023:SF3) |
| 2R A | AGAP013056 |  |
| 2R A | AGAP001225 |  |
| 2R A | AGAP001223 | ATP-DEPENDENT RNA HELICASE DDX55 (PTHR24031:SF2) |
| 2R A | AGAP001208 | ARGININE/SERINE-RICH COILED-COIL PROTEIN 2 (PTHR22426:SF2) |
| 2R A | AGAP013523 |  |
| 2R A | AGAP001214 | SOLUTE CARRIER FAMILY 35 MEMBER G1 (PTHR22911:SF6) |
| 2R A | AGAP001187 | HOMEOBOX PROTEIN CUT (PTHR14043:SF2) |
| 2R A | AGAP001205 | CHITIN SYNTHASE 2, ISOFORM D (PTHR22914:SF14) |
| 2R A | AGAP013012 |  |
| 2R A | AGAP001201 | DEFECTIVE PROBOSCIS EXTENSION RESPONSE 7, ISOFORM F (PTHR23279:SF6) |
| 2R A | AGAP001211 | LD44762P (PTHR47980:SF24) |
| 2R A | AGAP001213 |  |
| 2R A | AGAP001190 | AT20289P-RELATED (PTHR24276:SF78) |
| 2R A | AGAP001229 | DYNEIN LIGHT CHAIN TCTEX-TYPE (PTHR21255:SF4) |
| 2R A | AGAP001193 |  |
| 2R A | AGAP001228 | DNA-DIRECTED RNA POLYMERASE II SUBUNIT RPB4 (PTHR21297:SF0) |
| 2R A | AGAP013518 |  |
| 2R A | AGAP001198 |  |
| 2R A | AGAP001227 | GAMMA-TUBULIN COMPLEX COMPONENT 5 (PTHR19302:SF33) |
| 2R A | AGAP001194 | MICROTUBULE-ASSOCIATED PROTEIN FUTSCH (PTHR13843:SF12) |
| 2R A | AGAP013177 |  |
| 2R A | AGAP001224 | CLEAVAGE AND POLYADENYLATION SPECIFICITY FACTOR SUBUNIT 3 (PTHR11203:SF11) |
| 2R A | AGAP001192 |  |
| 2R A | AGAP001209 | UBIQUITIN CARBOXYL-TERMINAL HYDROLASE 32 (PTHR21646:SF46) |
| 2R A | AGAP001212 | PEPTIDOGLYCAN-RECOGNITION PROTEIN LB-RELATED (PTHR11022:SF67) |
| 2R A | AGAP013048 | GEO11133P1 (PTHR48024:SF12) |
| 2R A | AGAP001219 | TUBULIN-LIKE PROTEIN ALPHA-4B-RELATED (PTHR11588:SF239) |
| 2R A | AGAP001220 | NAD-DEPENDENT PROTEIN DEACETYLASE SIRTUIN-7 (PTHR45853:SF4) |
| 2R A | AGAP001222 | METHYLTRANSFERASE-LIKE PROTEIN 23 (PTHR14614:SF2) |
| 2R A | AGAP001203 | AGAP001203-PA (PTHR22933:SF19) |
| 2R B | AGAP001694 | EXOCYST COMPLEX COMPONENT 4 (PTHR14146:SF0) |
| 2R B | AGAP001680 | LD08718P (PTHR12876:SF35) |
| 2R B | AGAP001687 | RHO GTPASE-ACTIVATING PROTEIN 19 (PTHR14963:SF7) |
| 2R B | AGAP001695 | ZGC:112496 (PTHR21521:SF0) |
| 2R B | AGAP001688 | EXOSTOSIN-LIKE 3 (PTHR11062:SF73) |
| 2R B | AGAP001690 | RAB3 INTERACTING MOLECULE, ISOFORM F (PTHR12157:SF21) |
| 2R B | AGAP001681 | UBIQUITIN CONJUGATION FACTOR E4 A (PTHR13931:SF2) |
| 2R B | AGAP001684 | ALKALINE PHOSPHATASE-RELATED (PTHR11596:SF75) |
| 2R B | AGAP001686 | U6 SNRNA-ASSOCIATED SM-LIKE PROTEIN LSM1 (PTHR15588:SF8) |
| 2R B | AGAP001677 | LD24704P (PTHR10026:SF13) |
| 2R B | AGAP001682 | MITOCHONDRIAL INNER MEMBRANE PROTEASE SUBUNIT 1 (PTHR12383:SF16) |
| 2R B | AGAP001683 | PERIPHERAL PLASMA MEMBRANE PROTEIN CASK (PTHR23122:SF7) |
| 2R B | AGAP013145 | PROTEIN COUCH POTATO (PTHR10501:SF41) |
| 2R B | AGAP001685 | NUCLEAR PORE COMPLEX PROTEIN NUP107 (PTHR13003:SF2) |
| 2L | AGAP004812 | 85/88 KDA CALCIUM-INDEPENDENT PHOSPHOLIPASE A2 (PTHR24139:SF34) |
| 2L | AGAP004725 | EUKARYOTIC TRANSLATION INITIATION FACTOR 3 SUBUNIT C-RELATED (PTHR13937:SF0) |
| 2L | AGAP004686 | WW DOMAIN-CONTAINING ADAPTER PROTEIN WITH COILED-COIL (PTHR15911:SF6) |
| 2L | AGAP004798 | COATOMER SUBUNIT BETA' (PTHR19876:SF2) |
| 2L | AGAP004757 | PIRNA BIOGENESIS PROTEIN EXD1 (PTHR46628:SF1) |
| 2L | AGAP004823 | PROTEIN SPROUTY (PTHR12365:SF7) |
| 2L | AGAP004767 | TRANSCRIPTION FACTOR KEN (PTHR45993:SF7) |
| 2L | AGAP004729 | CHORION TRANSCRIPTION FACTOR CF2-RELATED (PTHR24388:SF77) |
| 2L | AGAP004806 | GALECTIN (PTHR11346:SF164) |
| 2L | AGAP004745 | TRANSCRIPTION ELONGATION REGULATOR HOMOLOG (PTHR15377:SF3) |
| 2L | AGAP004799 |  |
| 2L | AGAP004771 |  |
| 2L | AGAP004718 | RE21922P (PTHR11003:SF276) |
| 2L | AGAP004723 | FI06908P-RELATED (PTHR22812:SF151) |
| 2L | AGAP004827 | AGAP004827-PA (PTHR19871:SF28) |
| 2L | AGAP004699 | RAF HOMOLOG SERINE/THREONINE-PROTEIN KINASE RAF (PTHR44329:SF193) |
| 2L | AGAP004719 | EG:BACR7A4.3 PROTEIN-RELATED (PTHR24260:SF107) |
| 2L | AGAP004763 | FI02944P-RELATED (PTHR10174:SF216) |
| 2L | AGAP004687 | 6-PHOSPHOGLUCONATE DEHYDROGENASE, DECARBOXYLATING (PTHR11811:SF25) |
| 2L | AGAP004811 |  |
| 2L | AGAP004739 | H/ACA RIBONUCLEOPROTEIN COMPLEX SUBUNIT DKC1 (PTHR23127:SF0) |
| 2L | AGAP004807 | GALECTIN (PTHR11346:SF164) |
| 2L | AGAP004797 | AGAP004797-PA (PTHR22904:SF525) |
| 2L | AGAP004781 | BETA4GALNACTA (PTHR19300:SF52) |
| 2L | AGAP004810 |  |
| 2L | AGAP004819 | LP05237P-RELATED (PTHR12300:SF22) |
| 2L | AGAP004786 | PYRUVATE DEHYDROGENASE E1 COMPONENT SUBUNIT ALPHA (PTHR11516:SF55) |
| 2L | AGAP004747 | PRESEQUENCE PROTEASE, MITOCHONDRIAL (PTHR43016:SF13) |
| 2L | AGAP004724 | INTRAFLAGELLAR TRANSPORT PROTEIN 74 HOMOLOG (PTHR31432:SF0) |
| 2L | AGAP004780 | CYCLIN-DEPENDENT KINASE 12 (PTHR24056:SF233) |
| 2L | AGAP004791,AGAP004789 | HIGH MOBILITY GROUP PROTEIN 2 (PTHR46040:SF3) |
| 2L | AGAP004693 | NUCLEAR RECEPTOR SUBFAMILY 6 GROUP A MEMBER 1 (PTHR48092:SF18) |
| 2L | AGAP004762 | FI02944P-RELATED (PTHR10174:SF216) |
| 2L | AGAP004711 | ATP-DEPENDENT RNA HELICASE DDX41-RELATED (PTHR47958:SF103) |
| 2L | AGAP004733 | AGAP004733-PA (PTHR46105:SF5) |
| 2L | AGAP004758 | PROTEASOMAL UBIQUITIN RECEPTOR ADRM1 (PTHR12225:SF0) |
| 2L | AGAP004769 | RE63021P (PTHR24070:SF406) |
| 2L | AGAP004748 |  |
| 2L | AGAP004717 | FI03418P (PTHR11003:SF279) |
| 2L | AGAP004800 |  |
| 2L | AGAP004696 | HOMEOBOX PROTEIN EXTRADENTICLE (PTHR11850:SF108) |
| 2L | AGAP004749 | OXYGEN-DEPENDENT COPROPORPHYRINOGEN-III OXIDASE, MITOCHONDRIAL (PTHR10755:SF0) |
| 2L | AGAP004768 | PERIODIC TRYPTOPHAN PROTEIN 1 HOMOLOG (PTHR14091:SF0) |
| 2L | AGAP004813 |  |
| 2L | AGAP004737 | RHOMBOID-4 (PTHR45840:SF8) |
| 2L | AGAP004772 | SLIT-ROBO GAP HOMOLOG (PTHR14166:SF17) |
| 2L | AGAP004742 | PYRUVATE CARBOXYLASE, MITOCHONDRIAL (PTHR43778:SF2) |
| 2L | AGAP004752 | TRNA (GUANINE-N(7)-)-METHYLTRANSFERASE (PTHR23417:SF16) |
| 2L | AGAP004744 | SUCCINATE--COA LIGASE [ADP-FORMING] SUBUNIT BETA, MITOCHONDRIAL (PTHR11815:SF10) |
| 2L | AGAP004776 | PRE-MRNA-SPLICING FACTOR SLU7 (PTHR12942:SF2) |
| 2L | AGAP004770 | GH13245P2-RELATED (PTHR24276:SF83) |
| 2L | AGAP004759 | OLIGORIBONUCLEASE, MITOCHONDRIAL (PTHR11046:SF0) |
| 2L | AGAP004820 | RNA 3'-TERMINAL PHOSPHATE CYCLASE (PTHR11096:SF0) |
| 2L | AGAP004753 |  |
| 2L | AGAP004741 | AT07769P-RELATED (PTHR24260:SF90) |
| 2L | AGAP004814 |  |
| 2L | AGAP004703 | DNA-DIRECTED RNA POLYMERASE III SUBUNIT RPC1 (PTHR19376:SF32) |
| 2L | AGAP004704 | CXXC-TYPE ZINC FINGER PROTEIN 1 (PTHR46174:SF1) |
| 2L | AGAP004755 | VACUOLAR PROTEIN-SORTING-ASSOCIATED PROTEIN 36 (PTHR13128:SF12) |
| 2L | AGAP004715 | FI22513P1 (PTHR13847:SF257) |
| 2L | AGAP004712 |  |
| 2L | AGAP004691 | CHIP, ISOFORM B (PTHR10378:SF19) |
| 2L | AGAP004765 | ATP-DEPENDENT RNA HELICASE DDX1 (PTHR24031:SF307) |
| 2L | AGAP004722 | IRON-SULFUR CLUSTER CO-CHAPERONE PROTEIN HSCB (PTHR14021:SF15) |
| 2L | AGAP004692 | RAS GTPASE-ACTIVATING PROTEIN-RELATED (PTHR10194:SF125) |
| 2L | AGAP004764 | SNAIL FAMILY ZINC FINGER 2 TRANSCRIPTION FACTOR HOMOLOG (PTHR24409:SF375) |
| 2L | AGAP004802 | 4-HYDROXYPHENYLPYRUVATE DIOXYGENASE (PTHR11959:SF1) |
| 2L | AGAP004804 |  |
| 2L | AGAP004778 | BOLA-LIKE PROTEIN 3 (PTHR46188:SF1) |
| 2L | AGAP004790,AGAP004788 | ATP SYNTHASE MEMBRANE SUBUNIT DAPIT, MITOCHONDRIAL (PTHR34038:SF1) |
| 2L | AGAP004777 | AGAP004777-PA (PTHR21228:SF62) |
| 2L | AGAP004773 | PYRUVATE DEHYDROGENASE E1 COMPONENT SUBUNIT ALPHA (PTHR11516:SF55) |
| 2L | AGAP004720 | AGAP004720-PA (PTHR11003:SF263) |
| 2L | AGAP004815 |  |
| 2L | AGAP004794 | MIP10846P1-RELATED (PTHR11610:SF161) |
| 2L | AGAP004793 | ORNITHINE AMINOTRANSFERASE, MITOCHONDRIAL (PTHR11986:SF18) |
| 2L | AGAP004710 | CYTOCHROME B-C1 COMPLEX SUBUNIT 9 (PTHR12980:SF0) |
| 2L | AGAP004808 | AMINOPEPTIDASE (PTHR11533:SF283) |
| 2L | AGAP004805 | FORMIN-LIKE PROTEIN (PTHR45857:SF6) |
| 2L | AGAP004738 | PROTEIN RED (PTHR12765:SF5) |
| 2L | AGAP004761 | MULTIPLE WING HAIRS, ISOFORM C (PTHR45857:SF4) |
| 2L | AGAP004824 | EUKARYOTIC TRANSLATION INITIATION FACTOR 5B (PTHR43381:SF4) |
| 2L | AGAP004740 | AT07769P-RELATED (PTHR24260:SF90) |
| 2L | AGAP004795 | RNA POLYMERASE II ELONGATION FACTOR ELL (PTHR23288:SF17) |
| 2L | AGAP004731 | RH14732P (PTHR12253:SF34) |
| 2L | AGAP004736 | MITOCHONDRIAL RIBOSOME-ASSOCIATED GTPASE 1 (PTHR45782:SF4) |
| 2L | AGAP004766 | ENDOPHILIN-A (PTHR14167:SF89) |
| 2L | AGAP004709 | 39S RIBOSOMAL PROTEIN L18, MITOCHONDRIAL (PTHR12899:SF3) |
| 2L | AGAP004787 | FATTY ACYL-COA REDUCTASE (PTHR11011:SF24) |
| 2L | AGAP004784 | FATTY ACYL-COA REDUCTASE (PTHR11011:SF24) |
| 2L | AGAP004816 | PHOSPHATIDYLINOSITOL-GLYCAN BIOSYNTHESIS CLASS F PROTEIN-RELATED (PTHR43157:SF31) |
| 2L | AGAP004708 | ARGININE--TRNA LIGASE, CYTOPLASMIC-RELATED (PTHR11956:SF5) |
| 2L | AGAP004707 | SODIUM CHANNEL PROTEIN PARA (PTHR10037:SF288) |
| 2L | AGAP004756 | F-BOX/WD REPEAT-CONTAINING PROTEIN 4 (PTHR14381:SF1) |
| 2L | AGAP004706 |  |
| 2L | AGAP004690 | PUPAL CUTICLE PROTEIN C1B-LIKE PROTEIN (PTHR39068:SF5) |
| 2L | AGAP004785 | GH10083P-RELATED (PTHR10174:SF222) |
| 2L | AGAP004801 | HUNTINGTIN INTERACTING PROTEIN 1 (PTHR10407:SF15) |
| 2L | AGAP004694 | RE24423P (PTHR11161:SF22) |
| 2L | AGAP004818 | 28S RIBOSOMAL PROTEIN S16, MITOCHONDRIAL (PTHR12919:SF27) |
| 2L | AGAP004689 | LD40707P (PTHR13055:SF12) |
| 2L | AGAP004817 | PROTEIN LINGERER (PTHR16308:SF13) |
| 2L | AGAP004700 | FI18411P1-RELATED (PTHR24260:SF91) |
| 2L | AGAP004726 | AGAP004726-PA (PTHR10559:SF18) |
| 2L | AGAP004716 |  |
| 2L | AGAP004774 | HOST CELL FACTOR (PTHR46003:SF1) |
| 2L | AGAP004713 |  |
| 2L | AGAP004825 | BCDNA.LD24702 (PTHR13030:SF14) |
| 2L | AGAP004792 | STAR-RELATED LIPID TRANSFER PROTEIN 7, MITOCHONDRIAL (PTHR19308:SF8) |
| 2L | AGAP004775 | XAA-PRO DIPEPTIDASE (PTHR43226:SF1) |
| 2L | AGAP004754 | CASPASE DRONC (PTHR10454:SF196) |
| 2L | AGAP004714 | CROL ALPHA (PTHR23226:SF176) |
| 2L | AGAP013546 | VACUOLAR ATPASE ASSEMBLY INTEGRAL MEMBRANE PROTEIN VMA21 HOMOLOG (PTHR31792:SF6) |
| 2L | AGAP004782 | TUBULIN-SPECIFIC CHAPERONE D (PTHR12658:SF0) |
| 2L | AGAP004743 | TRANSMEMBRANE EMP24 DOMAIN-CONTAINING PROTEIN 2 (PTHR22811:SF177) |
| 2L | AGAP004728 | ARMADILLO REPEAT-CONTAINING PROTEIN 2 (PTHR21356:SF1) |
| 2L | AGAP004809 | AMINOPEPTIDASE (PTHR11533:SF283) |
| 2L | AGAP004695 | MULTIVESICULAR BODY SUBUNIT 12A (PTHR31612:SF2) |
| 3L A | AGAP010820 |  |
| 3L A | AGAP010803 |  |
| 3L A | AGAP010787 | PROTEIN TIMELESS HOMOLOG (PTHR22940:SF4) |
| 3L A | AGAP010790 | CYTOPLASMIC DYNEIN 2 HEAVY CHAIN 1 (PTHR10676:SF352) |
| 3L A | AGAP010793 | SYNEMBRYN (PTHR12425:SF5) |
| 3L A | AGAP010809 | CHAOPTIN-LIKE PROTEIN (PTHR45617:SF82) |
| 3L A | AGAP010814 | GH01829P-RELATED (PTHR11412:SF136) |
| 3L A | AGAP010789 | LD24340P (PTHR39069:SF4) |
| 3L A | AGAP010784 | PROTEIN O-MANNOSYL-TRANSFERASE 1-RELATED (PTHR10050:SF51) |
| 3L A | AGAP010812 | GH01829P-RELATED (PTHR11412:SF136) |
| 3L A | AGAP010798 | GH13245P2-RELATED (PTHR24276:SF83) |
| 3L A | AGAP010792 | NADH DEHYDROGENASE [UBIQUINONE] 1 ALPHA SUBCOMPLEX SUBUNIT 9, MITOCHONDRIAL (PTHR12126:SF11) |
| 3L A | AGAP010777 | HUNCHBACK-LIKE (PTHR24392:SF33) |
| 3L A | AGAP010811 | GH05177P-RELATED (PTHR19143:SF383) |
| 3L A | AGAP013327 | PEROXIDASE (PTHR11475:SF86) |
| 3L A | AGAP010817 | AGAP006059-PA (PTHR47537:SF3) |
| 3L A | AGAP010818 | GH01829P-RELATED (PTHR11412:SF136) |
| 3L A | AGAP010783 | GEO02292P1-RELATED (PTHR23301:SF79) |
| 3L A | AGAP010799 |  |
| 3L A | AGAP010813 |  |
| 3L A | AGAP010786 | RNA (RNA) POLYMERASE II ASSOCIATED PROTEIN HOMOLOG (PTHR45984:SF1) |
| 3L A | AGAP010816 | GH01829P-RELATED (PTHR11412:SF136) |
| 3L A | AGAP010802 | COREPRESSOR OF PANGOLIN, ISOFORM A-RELATED (PTHR12243:SF47) |
| 3L A | AGAP010780 |  |
| 3L A | AGAP010782 | RAL GUANINE NUCLEOTIDE DISSOCIATION STIMULATOR-LIKE, ISOFORM E (PTHR23113:SF312) |
| 3L A | AGAP010800 | ZINC FINGER CCCH DOMAIN-CONTAINING PROTEIN 18 (PTHR46582:SF1) |
| 3L A | AGAP010807 | TRNA PSEUDOURIDINE(38/39) SYNTHASE (PTHR11142:SF5) |
| 3L A | AGAP010819 | GH01829P-RELATED (PTHR11412:SF136) |
| 3L A | AGAP010785 | FATTY ACYL-COA REDUCTASE (PTHR11011:SF81) |
| 3L A | AGAP010805 |  |
| 3L A | AGAP010806 | BCDNA.GH04802 (PTHR11003:SF158) |
| 3L A | AGAP010808 | ARC, ISOFORM A (PTHR19964:SF69) |
| 3L A | AGAP010776 | WHIRLIN (PTHR23116:SF37) |
| 3L A | AGAP010791 | POLYRIBONUCLEOTIDE NUCLEOTIDYLTRANSFERASE 1, MITOCHONDRIAL (PTHR11252:SF0) |
| 3L A | AGAP010794 | FI23527P1 (PTHR45820:SF4) |
| 3L A | AGAP010801 | PHOSPHATIDYLINOSITOL 4-KINASE ALPHA-RELATED (PTHR10048:SF15) |
| 3L A | AGAP010804 | SI:CH73-302O18.2 (PTHR45752:SF27) |
| 3L B | AGAP011332 | FINGER PUTATIVE TRANSCRIPTION FACTOR FAMILY-RELATED (PTHR24399:SF23) |
| 3L B | AGAP011316 | SENTRIN-SPECIFIC PROTEASE 8 (PTHR46468:SF1) |
| 3L B | AGAP011345 | GROWTH ARREST AND DNA DAMAGE-INDUCIBLE PROTEINS-INTERACTING PROTEIN 1 (PTHR31761:SF1) |
| 3L B | AGAP011320 | SUBFAMILY NOT NAMED (PTHR24230:SF141) |
| 3L B | AGAP011387 | MITOCHONDRIAL DIVISION PROTEIN 1-RELATED (PTHR19857:SF8) |
| 3L B | AGAP011323 | TCF3 FUSION PARTNER (PTHR35084:SF1) |
| 3L B | AGAP011361 | CHECKPOINT PROTEIN (PTHR12900:SF0) |
| 3L B | AGAP011330 | GLUTAREDOXIN-LIKE PROTEIN C5ORF63 HOMOLOG (PTHR33558:SF1) |
| 3L B | AGAP011317 |  |
| 3L B | AGAP011374 | ADAPTOR PROTEIN COMPLEX 1, MU SUBUNIT (PTHR10529:SF262) |
| 3L B | AGAP011365 | CARBOXYLIC ESTER HYDROLASE (PTHR43142:SF1) |
| 3L B | AGAP011377 |  |
| 3L B | AGAP011389 | PROTEIN CBG05349 (PTHR15192:SF8) |
| 3L B | AGAP011376 |  |
| 3L B | AGAP011362 | GATA ZINC FINGER DOMAIN-CONTAINING PROTEIN 1 (PTHR13340:SF2) |
| 3L B | AGAP011329 | ACETYL-COA ACETYLTRANSFERASE, MITOCHONDRIAL (PTHR18919:SF156) |
| 3L B | AGAP011356 | AMYLOID PROTEIN-BINDING PROTEIN 2 (PTHR46575:SF1) |
| 3L B | AGAP011368 | AGAP011368-PA (PTHR21066:SF17) |
| 3L B | AGAP011352 | GH12731P-RELATED (PTHR10937:SF0) |
| 3L B | AGAP011338 | GDP-FUCOSE PROTEIN O-FUCOSYLTRANSFERASE 1 (PTHR21420:SF3) |
| 3L B | AGAP011349 | GAMMA-AMINOBUTYRIC ACID RECEPTOR ALPHA-LIKE (PTHR18945:SF835) |
| 3L B | AGAP011363 | RAS-RELATED PROTEIN RAB6 (PTHR24073:SF352) |
| 3L B | AGAP011326 | DEATH-ASSOCIATED INHIBITOR OF APOPTOSIS 2 (PTHR10044:SF139) |
| 3L B | AGAP011372 |  |
| 3L B | AGAP011367 |  |
| 3L B | AGAP011321 | PROTOPORPHYRINOGEN OXIDASE (PTHR42923:SF3) |
